# Beyond teacher burnout: teacher emotional regulation mechanisms underlying learner engagement in English classrooms

**DOI:** 10.3389/fpsyg.2026.1952024

**Published:** 2026-09-21

**Authors:** Jing Sun

**Affiliations:** Department of Foreign Languages, Lyuliang University, Lyuliang, Shanxi, China

**Keywords:** educational psychology, emotional regulation, English language teaching, learner engagement, structural equation modeling, student engagement, teacher burnout, teacher wellbeing

## Abstract

**Background:**

Teacher burnout is an increasing psychological challenge in educational settings, affecting teachers’ emotional wellbeing and students’ learning experiences.

**Aim:**

This study examined the psychological mechanisms through which teacher burnout influences learner engagement in English language classrooms, with particular attention to the role of emotional regulation.

**Framework:**

Drawing on emotion regulation theory and educational psychology perspectives, this study explored whether teachers’ ability to manage emotional demands can reduce the negative impact of burnout on students’ classroom involvement.

**Method:**

Data were collected in 2025 from 428 English language teachers and 1,284 students from secondary and higher education institutions. Teacher burnout was assessed through emotional exhaustion, depersonalisation, and reduced professional efficacy; emotional regulation was measured through cognitive reappraisal and emotional control strategies; learner engagement was evaluated through behavioral participation, emotional involvement, and cognitive engagement indicators. Structural equation modelling was used to test direct and indirect relationships while controlling for relevant contextual factors.

**Findings:**

Teacher burnout significantly predicted lower learner engagement (*β* = −0.36, *p* < 0.001), whereas emotion regulation showed a positive association with student engagement (*β* = 0.42, *p* < 0.001). Mediation analysis indicated that emotion regulation partially mediated the adverse effects of burnout, highlighting its role in classroom interactions.

**Implications:**

Improving teachers’ emotional regulation skills and reducing occupational stress should be important priorities in educational policy. Integrating psychological support, emotional skills training, and burnout prevention initiatives may enhance teachers’ wellbeing and promote more engaging English language learning environments.

## 1 Introduction

Teacher burnout has emerged as a critical psychological concern within contemporary educational systems, affecting teachers’ emotional wellbeing, professional functioning, and quality of classroom interactions. Teaching requires continuous emotional involvement, effective communication, and the ability to respond constructively to diverse students’ needs. In English language classrooms, these demands become particularly complex because successful learning depends heavily on interaction, motivation, confidence, and supportive teacher–student relationships (Lagos et al., 2026). When teachers experience prolonged emotional exhaustion, excessive workloads, or reduced professional efficacy, their ability to maintain enthusiasm, provide emotional support, and create engaging learning environments may decline. Although burnout has traditionally been viewed as an individual occupational problem affecting teachers’ health and performance, recent psychological perspectives suggest that its consequences may extend beyond teachers and influence students’ learning behaviours, emotional experiences, and engagement in classroom activities (Li et al., 2025a).

Learner engagement is a central psychological factor associated with successful educational outcomes, particularly in language learning contexts where active participation and sustained cognitive involvement are essential. Engagement reflects students’ behavioural participation, emotional connection, and cognitive investment in learning tasks (Liu et al., 2026). Teachers significantly contribute to shaping these dimensions through their instructional behaviour, emotional expression, and classroom management approaches. However, teachers experiencing burnout may have limited emotional resources to encourage participation, respond patiently to students’ difficulties, and maintain positive classroom interactions (Guzmán-Martínez et al., 2026). Reduced emotional availability may indirectly influence students’ willingness to participate, collaborate, and invest effort in language learning activities (Yao et al., 2026). Therefore, understanding the psychological processes that connect teachers’ emotional experiences with learner engagement is essential for developing more effective educational interventions.

Emotion regulation has recently gained attention as an important psychological resource that may explain the differences in teachers’ responses to occupational stress. According to emotion regulation theory, individuals differ in their ability to monitor, modify, and manage their emotional responses according to situational demands. Specifically, Gross's (1998) process model of emotion regulation distinguishes between antecedent-focused strategies (such as cognitive reappraisal) and response-focused strategies (such as expressive suppression), with reappraisal consistently associated with more adaptive outcomes in occupational settings. In teaching contexts, cognitive reappraisal enables educators to reframe challenging classroom situations in less threatening ways, thereby preserving emotional resources that would otherwise be depleted by stress (Guzmán-Martínez et al., 2026; Wang et al., 2026). This theoretical perspective suggests that teachers with stronger reappraisal abilities may maintain effective classroom functioning despite experiencing burnout. Teachers with effective emotion regulation skills may be better able to control frustration, recover from stressful classroom situations, and maintain supportive interactions despite facing professional challenges. In contrast, teachers with limited regulatory capacity may experience greater difficulty in managing emotional demands, which can intensify the negative effects of burnout on classroom relationships. Thus, emotion regulation may function as an explanatory mechanism that reduces the harmful influence of burnout and supports more positive learning environments. This study is grounded in two complementary theoretical frameworks. First, Gross's (1998) process model of emotion regulation provides a foundation for understanding how teachers’ cognitive reappraisal and expressive suppression strategies influence their ability to manage classroom stress. This model proposes that individuals who employ antecedent-focused strategies, such as reappraisal, are better able to modify emotional responses before they become fully activated, thereby preserving psychological resources that would otherwise be depleted by prolonged stress exposure. Second, self-determination theory (SDT; Deci and Ryan, 2000) offers a complementary lens for understanding student outcomes of teachers’ emotional processes. SDT posits that students’ engagement is shaped by their perceptions of autonomy, competence, and relatedness in the classroom. Teachers with stronger emotion regulation abilities are better positioned to support these basic psychological needs through warm, responsive, and autonomy-supportive interactions, even when they experience occupational stress. The integration of these frameworks suggests that emotion regulation may serve as a bridging mechanism: burnout depletes teachers’ regulatory resources, which in turn compromises their capacity to support students’ psychological needs, ultimately reducing learner engagement.

Despite the increasing research on teacher burnout and emotional wellbeing, several important limitations remain. Previous studies have primarily focused on identifying the causes and consequences of burnout among teachers, including job stress, workload, reduced satisfaction, and professional withdrawal (Solhi et al., 2026). However, this body of research has predominantly treated burnout as an individual teacher outcome rather than examining its relational consequences for students. For instance, while studies have documented how burnout affects teachers’ health, job satisfaction, and career intentions (Li et al., 2025a; Solhi et al., 2026), significantly less attention has been directed toward understanding the psychological processes that transmit teachers’ emotional difficulties into reduced student engagement. This represents a critical gap because educational theories consistently emphasize that learning is fundamentally an interpersonal and emotionally mediated process (Guzmán-Martínez et al., 2026; Lagos et al., 2026). Although these studies provide valuable insights, they often overlook the mechanisms through which teacher burnout influences students’ psychological and behavioural outcomes. Specifically, the existing literature lacks a clear theoretical explanation of how teachers’ internal emotional states translate into observable changes in classroom dynamics and student participation. Recent educational psychology perspectives have called for more process-oriented research that moves beyond identifying correlations to examining the mediating mechanisms that link teacher wellbeing with student outcomes (France and Harkins-Brown, 2026; Guzmán-Martínez et al., 2026). In particular, limited attention has been given to the mediating role of emotional regulation in explaining how teachers’ internal emotional states translate into changes in learner engagement (Xu et al., 2026). Although theoretical work has suggested that teachers’ ability to regulate emotions may be a crucial resource for maintaining effective classroom interactions, empirical studies directly testing this mediation pathway remain scarce. This is particularly notable given that emotion regulation theory explicitly proposes that individual differences in regulatory capacity influence how people manage and respond to stressful experiences (Wang et al., 2026), making it a theoretically plausible mechanism for understanding the burnout-engagement link. Furthermore, research examining these relationships within English language classrooms remains relatively limited, despite the strong emotional and interpersonal demands associated with language teaching. This gap highlights the need for a more integrated psychological framework that considers teacher burnout, emotional regulation, and learner engagement as interconnected processes.

To address this gap, the present study addresses the following research questions:

*RQ1*: Is teacher burnout significantly associated with learner engagement in English language classrooms?

*RQ2*: Is teacher emotion regulation significantly associated with learner engagement?

*RQ3*: Does emotion regulation mediate the relationship between teacher burnout and learner engagement, such that burnout is associated with reduced regulatory capacity, which in turn is associated with lower student engagement?

Based on the theoretical framework and prior empirical evidence, the following hypotheses were tested:

*H1*: Teacher burnout will be negatively associated with learner engagement.

*H2*: Teacher emotion regulation will be positively associated with learner engagement.

*H3*: Emotion regulation will mediate the relationship between teacher burnout and learner engagement, such that the indirect effect through emotion regulation will be significant.

To address this gap, the present study proposes and examines a psychological model examining how teacher burnout is associated with learner engagement through an emotional regulation pathway in English language classrooms (Gan et al., 2026). By combining perspectives from educational psychology and emotion regulation theory, this study moves beyond viewing burnout as only a negative teacher outcome and investigates its broader implications for classroom learning processes. Using data from English language teachers and students, the study evaluates the association between teacher burnout and learner engagement and examines whether emotion regulation functions as a potential mediating mechanism in this relationship.(Dou et al., 2026). This approach provides a deeper understanding of the emotional dynamics underlying classroom engagement.

The contribution of this study is threefold. First, it expands existing burnout research by shifting attention from teacher-centered outcomes toward the psychological effects of teachers’ emotional experiences on students’ learning engagement. Second, it identifies emotion regulation as a key mechanism that explains how burnout shapes classroom participation and involvement, providing a more comprehensive understanding of teacher–student emotional processes. Third, the findings offer practical implications for educational institutions by emphasizing the importance of emotional skills development, psychological support systems, and burnout prevention strategies in teacher training programs (Fu et al., 2026). By highlighting the role of teachers’ emotional resources in creating engaging learning environments, this study contributes to the broader field of educational psychology and provides evidence-based directions for improving both teacher wellbeing and student engagement in English language classrooms.

## 2 Literature review

### 2.1 Teacher burnout as a psychological challenge in education

Teacher burnout has become an important psychological issue within educational settings because of its influence on teachers’ emotional health, professional performance, and classroom interactions. Unlike temporary work-related stress, burnout develops through prolonged exposure to occupational pressures and is commonly reflected in emotional exhaustion, reduced professional efficacy, and psychological distancing from teaching responsibilities. Teachers are required to manage multiple demands simultaneously, including lesson preparation, classroom management, administrative responsibilities, and continuous emotional interactions with students. Over time, these demands may reduce teachers’ psychological resources and make it more difficult to maintain enthusiasm, motivation, and positive relationships within the classroom. The impact of teacher burnout is particularly relevant in English language classrooms, where effective learning depends strongly on communication, interaction, and supportive teacher–student relationships. Teachers experiencing emotional exhaustion may have limited capacity to provide encouragement, respond patiently to students’ difficulties, and create an engaging learning atmosphere (Ma and Li, 2026). As a result, burnout may influence not only teachers’ personal wellbeing but also students’ learning experiences and classroom participation.

Previous research has primarily examined teacher burnout in relation to individual outcomes, such as job satisfaction, professional commitment, and psychological distress. For example, meta-analytic evidence has consistently linked teacher burnout with reduced job performance, increased absenteeism, and higher turnover intentions (Li et al., 2025a; Solhi et al., 2026). However, these studies have largely adopted a within-teacher perspective, leaving the interpersonal consequences of burnout relatively unexplored. More recently, researchers have begun to recognize that teachers’ emotional wellbeing may have cascading effects on students’ academic experiences (France and Harkins-Brown, 2026; Ma and Li, 2026). This shift toward relational perspectives represents an important theoretical development, yet the specific pathways through which burnout influences student outcomes remain insufficiently specified. However, increasing evidence suggests that burnout may also affect broader educational processes by shaping classroom climate and teacher behaviours. Understanding burnout as a psychological phenomenon provides deeper insight into how teachers’ emotional conditions influence student engagement and learning experiences. Therefore, examining teacher burnout within a classroom context is essential for identifying psychological mechanisms that may protect both teacher wellbeing and student involvement in learning activities. Several theoretical perspectives help explain why burnout may have interpersonal consequences. The conservation of resources (COR) theory (Hobfoll, 1989) proposes that individuals strive to protect their existing resources and acquire new ones. When teachers experience chronic stress that depletes their emotional resources, they enter a state of resource loss that makes it increasingly difficult to invest in emotionally demanding interactions with students. This resource depletion perspective suggests that burnout does not merely affect teachers’ internal states but fundamentally alters their capacity for relational engagement, which has direct implications for students’ classroom experiences. Moreover, emotional contagion theory (Hatfield et al., 1994) suggests that emotions can transfer between individuals in social interactions; teachers experiencing burnout may unintentionally transmit negative emotional states to students through verbal and nonverbal cues, thereby reducing students’ emotional engagement and willingness to participate.

### 2.2 Emotion regulation as a psychological resource

Emotion regulation refers to the ability to monitor and modify emotional responses according to situational demands. For teachers, effective emotion regulation is essential for managing classroom stress, maintaining supportive interactions, and responding constructively to challenging situations. Strategies such as cognitive reappraisal and emotional control help teachers preserve emotional balance and sustain positive relationships with students. Research indicates that teachers with stronger emotion regulation skills demonstrate greater resilience, improved classroom management, and enhanced professional effectiveness (Research and Teaching, 2026; Quarterly, 2026). However, the literature is not entirely consistent regarding the nature and effects of emotion regulation in teaching contexts. While cognitive reappraisal has been consistently associated with positive outcomes, some research suggests that emotional suppression may have paradoxical effects, reducing visible stress expressions while increasing internal psychological strain (Wang et al., 2026; Yao et al., 2026). Moreover, studies have shown that the effectiveness of emotion regulation strategies may vary across cultural contexts and teaching situations (Lagos et al., 2026). These divergent findings underscore the need for further research examining emotion regulation processes in specific educational settings, such as English language classrooms, where emotional demands may be particularly pronounced. Moreover, emotion regulation may reduce the negative consequences of occupational stress by protecting teachers’ emotional resources. Thus, it represents an important psychological mechanism that may explain why some teachers maintain effective classroom functioning despite experiencing professional pressures. The mechanisms through which emotion regulation supports teaching effectiveness are multifaceted. First, cognitive reappraisal enables teachers to reinterpret challenging classroom situations—such as student disengagement or disruptive behaviour—in less threatening ways, thereby reducing the emotional intensity of these encounters and preserving energy for instructional activities (Sutton and Wheatley, 2003). Second, effective emotion regulation helps teachers maintain emotional authenticity while also displaying appropriate emotional expressions that support student learning, a process referred to as “emotional labor” in teaching contexts (Hochschild, 1983). Third, teachers with stronger regulatory abilities are better equipped to recover from stressful interactions and maintain positive emotional states across the school day, which contributes to more consistent and supportive classroom climates (Jennings and Greenberg, 2009). These mechanisms suggest that emotion regulation is not merely a personal coping strategy but a professional competency that directly influences teaching effectiveness and classroom quality.

### 2.3 Teacher emotions and learner engagement

Learner engagement is a fundamental psychological component of successful learning, reflecting the extent to which students participate actively, develop emotional connections, and invest cognitive effort in educational activities. In English language classrooms, engagement becomes particularly important because language development depends heavily on communication, interaction, confidence, and continuous involvement in classroom tasks. Unlike subjects where learning may occur through passive observation, language acquisition requires students to express ideas, participate in discussions, practice new skills, and engage in meaningful exchanges with teachers and peers (Wei and Wang, 2026). Therefore, the emotional quality of the classroom environment plays an important role in shaping students’ willingness to participate and persist in learning activities. Teachers are central contributors to the development of learner engagement because their emotional expressions, instructional behaviours, and interpersonal relationships influence how students perceive the learning environment. Self-determination theory (SDT) provides a useful framework for understanding this process, as it proposes that students’ engagement is shaped by their perceptions of autonomy, competence, and relatedness in the classroom (Gan et al., 2026; Wei and Wang, 2026). Teachers experiencing burnout may struggle to support these basic psychological needs due to depleted emotional resources, reduced patience, and diminished capacity for warm, responsive interactions. Conversely, emotionally well-regulated teachers are better positioned to provide the autonomy support, constructive feedback, and emotional connection that foster student engagement. Teachers who demonstrate enthusiasm, patience, and emotional support are more likely to encourage students’ confidence and create a sense of psychological safety. Such environments allow learners to participate without fear of failure and increase their motivation to invest effort in language learning. In contrast, teachers experiencing emotional difficulties may unintentionally create less supportive classroom conditions.

Teacher burnout, particularly emotional exhaustion, can negatively influence the quality of teacher–student interactions. When teachers experience prolonged psychological strain, they may have reduced emotional availability, limited patience, and decreased capacity to provide individualized encouragement and constructive feedback (Li et al., 2025b). These changes may affect students’ perceptions of classroom support and reduce their behavioural participation, emotional connection, and cognitive involvement. However, the relationship between teacher emotions and learner engagement is not simply determined by the presence of stress but also by teachers’ ability to regulate and manage emotional responses. Recent educational psychology perspectives emphasize that teachers’ emotional experiences represent an important pathway through which classroom processes develop. Understanding how teacher emotions influence learner engagement provides valuable insight into improving classroom effectiveness. Therefore, examining the connection between teacher burnout, emotional regulation, and student engagement is essential for developing psychologically supportive English language learning environments.

### 2.4 Emotion regulation as a link between burnout and engagement

Although previous research has provided important evidence regarding the relationship between teacher burnout and educational outcomes, the psychological processes explaining how burnout influences students’ learning experiences remain insufficiently understood. Several studies have documented the negative association between teacher burnout and various student outcomes, including academic achievement, classroom behaviour, and motivation (Boroughani et al., 2025; France and Harkins-Brown, 2026). However, the mechanisms underlying these associations remain largely speculative. Some researchers have proposed that burnout reduces teachers’ instructional quality (Ma and Li, 2026), while others have emphasized the role of emotional availability and teacher-student relationship quality (Wei and Wang, 2026). The present study extends this work by specifically examining emotion regulation as a mediating mechanism that explains how burnout affects teachers’ capacity to maintain supportive classroom interactions. Emotion regulation may represent an important mechanism that connects teachers’ emotional challenges with learner engagement (Wang et al., 2026). Teachers experiencing high levels of burnout often face difficulties in managing frustration, maintaining emotional balance, and responding effectively to classroom demands. These emotional difficulties may reduce the quality of teacher–student interactions and limit teachers’ ability to provide encouragement, constructive feedback, and psychological support. In contrast, teachers with stronger emotion regulation abilities may be better equipped to manage stressful situations and maintain positive classroom relationships despite professional pressures. Through strategies such as cognitive reappraisal and emotional control, teachers can modify negative emotional responses and respond to classroom challenges in more adaptive ways. This ability may help protect teachers from the harmful consequences of burnout and preserve an engaging learning environment for students.

From an educational psychology perspective, emotion regulation is not only an individual psychological skill but also a relational process that influences classroom dynamics. Teachers’ emotional responses can shape students’ perceptions of support, motivation, and belonging, which are essential components of learner engagement (France and Harkins-Brown, 2026). Therefore, examining emotion regulation as a pathway between burnout and engagement provides a deeper understanding of how teachers’ internal emotional processes influence student participation and learning experiences. This perspective extends existing burnout research by highlighting the importance of emotional resources in maintaining effective and supportive English language classrooms.

### 2.5 Research gap and present study framework

Existing research has substantially contributed to understanding teacher burnout and its consequences for educational outcomes; however, important questions remain regarding the psychological processes that explain how teachers’ emotional experiences influence student engagement. Previous studies have largely focused on identifying the causes of burnout and its effects on teachers’ professional wellbeing, job satisfaction, and performance. Although these findings provide valuable knowledge, less attention has been given to the mechanisms through which teacher burnout affects students’ classroom experiences, particularly in English language learning contexts where emotional interaction and communication are central to successful learning. A major limitation in the existing literature is the insufficient examination of emotion regulation as a protective psychological mechanism linking teacher burnout with learner engagement. While burnout may reduce teachers’ emotional resources and weaken classroom interactions, effective emotion regulation may help teachers manage professional pressures and maintain supportive learning environments. Understanding this process is essential because teachers’ emotional responses not only influence their own psychological functioning but also shape students’ motivation, participation, and involvement in classroom activities.

To address this gap, the present study develops a psychological framework that examines the relationship between teacher burnout, emotion regulation, and learner engagement in English language classrooms. By integrating educational psychology and emotion regulation perspectives, this research moves beyond viewing burnout as only a negative occupational outcome and explores how teachers’ emotional resources can influence student learning experiences. The study provides a more comprehensive explanation of teacher–student emotional dynamics by investigating whether emotion regulation reduces the negative impact of burnout on learner engagement (Guzmán-Martínez et al., 2026). The findings of this research may provide valuable implications for educational institutions by highlighting the importance of emotional skills development, teacher wellbeing initiatives, and burnout prevention strategies. Strengthening teachers’ emotional resources may contribute to healthier classroom environments and promote sustained learner engagement in English language education.

## 3 Materials and methods

### 3.1 Research design and participants

This study adopted a quantitative research design to examine the psychological processes linking teacher burnout, emotional regulation, and learner engagement in English language classrooms. The research was based on the assumption that teachers’ emotional experiences influence classroom interactions and may subsequently shape students’ willingness to participate, remain motivated, and engage deeply in learning activities. A cross-sectional approach was selected because it allowed the investigation of relationships among psychological constructs within naturally occurring educational settings. Data were collected during the 2025 academic year from secondary and higher education institutions where English was taught as a formal subject. The final sample included 428 English language teachers and 1,284 students. Teachers represented different levels of teaching experience and institutional backgrounds, allowing the study to capture variation in professional demands and classroom contexts. Each teacher was associated with a group of students from their classroom to establish a direct connection between teacher-related psychological factors and learner engagement outcomes. This nested structure—with students (Level 1) clustered within teachers/classrooms (Level 2)—creates dependencies in the data that must be accounted for in statistical analyses. Specifically, students within the same classroom are likely to share common experiences, instructional approaches, and classroom climate characteristics, violating the assumption of independent observations required for conventional single-level analysis. To address this, we estimated intraclass correlation coefficients (ICCs) for learner engagement to determine the proportion of variance attributable to classroom-level differences. The ICC for learner engagement was 0.28, indicating that approximately 28% of the variance in student engagement existed between classrooms. This substantiates the need for analytical procedures that account for the hierarchical data structure.

Participation was voluntary, and all participants were informed about the objectives of the study before completing the questionnaires. Ethical principles, including informed consent, confidentiality, and the protection of participant information, were followed throughout the research process. No personally identifiable information was collected, and participants were allowed to withdraw from the study at any stage.

The sample size was considered appropriate for structural equation modeling because it provided sufficient statistical power for examining complex relationships among latent psychological variables. Given the nested data structure, we employed multilevel structural equation modeling (MSEM) using Mplus 8.0 to test the hypothesized relationships. MSEM allows for the simultaneous estimation of within- and between-group effects while accounting for non-independence of observations. Teacher-level variables (burnout and emotion regulation) were modeled at the between level (Level 2), while student-level engagement was modeled at the within level (Level 1), with classroom-level aggregation of student engagement serving as the between-level outcome. The design enabled the study to move beyond examining burnout as an individual teacher problem and instead explore how teachers’ emotional experiences influence the broader classroom learning environment.

### 3.2 Study variables and measurement instruments

The present study examined three central psychological constructs that represent the proposed relationship between teachers’ emotional experiences and students’ classroom engagement: teacher burnout, emotion regulation, and learner engagement. These variables were selected based on educational psychology perspectives suggesting that teachers’ internal emotional processes can influence classroom interactions and student learning behaviours. Teacher burnout was treated as the primary predictor variable and was conceptualized as a multidimensional psychological condition involving emotional exhaustion, depersonalization, and reduced professional efficacy (Boroughani et al., 2025). Emotional exhaustion reflects teachers’ feelings of emotional and psychological fatigue resulting from continuous professional demands, whereas depersonalization represents emotional distancing from students and teaching responsibilities. Reduced professional efficacy refers to teachers’ perceptions of declining competence and effectiveness in their instructional roles.

Emotion regulation was examined as a mediating psychological mechanism explaining how teachers manage emotional demands within classroom environments. It was assessed through two key dimensions: cognitive reappraisal and expressive suppression. Cognitive reappraisal reflects the ability to reinterpret stressful experiences in adaptive ways, while emotional control represents the capacity to regulate emotional expressions and maintain professional responses during challenging classroom situations. Learner engagement was considered the primary outcome variable and was evaluated through three dimensions: behavioural, emotional, and cognitive engagement. Behavioural engagement captures students’ participation and involvement in classroom activities, emotional engagement reflects students’ interest and psychological connection with learning, and cognitive engagement represents students’ mental effort and active use of learning strategies (Xu et al., 2026).

In addition to the main psychological variables, teaching experience, workload, and classroom characteristics were included as control variables to account for contextual differences that may influence learner engagement. All constructs were measured using established psychological and educational assessment instruments adapted to the English language classroom context. Teacher burnout was assessed using the Maslach Burnout Inventory-Educators Survey (MBI-ES; Maslach et al., 1996), a 22-item instrument measuring three dimensions: emotional exhaustion (9 items; e.g., “I feel emotionally drained from my work”), depersonalization (5 items; e.g., “I feel I treat some students as if they were impersonal objects”), and reduced personal accomplishment (8 items; e.g., “I feel I am positively influencing students’ lives through my work”). Responses were recorded on a 7-point Likert scale ranging from 0 (never) to 6 (every day). The Chinese version of the MBI-ES has demonstrated acceptable reliability and validity in previous studies with Chinese teachers (Li et al., 2025a).

Emotion regulation was measured using the Emotion Regulation Questionnaire (ERQ; Gross and John, 2003), a 10-item instrument assessing two strategies: cognitive reappraisal (6 items; e.g., “I control my emotions by changing the way I think about the situation I’m in”) and expressive suppression (4 items; e.g., “I control my emotions by not expressing them”). Responses were rated on a 7-point Likert scale from 1 (strongly disagree) to 7 (strongly agree). The ERQ has been widely used in educational research and validated in Chinese populations (Wang et al., 2026).

Learner engagement was assessed using the Student Engagement Scale (SES; Fredricks et al., 2004), adapted for English language classroom contexts. The scale comprises 17 items measuring three dimensions: behavioural engagement (5 items; e.g., “I pay attention in English class”), emotional engagement (6 items; e.g., “I enjoy learning English”), and cognitive engagement (6 items; e.g., “I try to connect what I am learning in English class to things I have learned before”). Students responded on a 5-point Likert scale ranging from 1 (strongly disagree) to 5 (strongly agree). The Chinese adaptation of this scale has demonstrated strong psychometric properties in previous research (Xu et al., 2026).

All instruments were administered in Chinese, with translation and back-translation procedures conducted following established guidelines to ensure conceptual equivalence. Prior to data collection, a pilot study with 30 teachers and 30 students was conducted to verify item clarity and appropriateness for the target population. Minor wording adjustments were made based on pilot feedback. Prior to structural analysis, reliability and validity assessments were conducted to confirm that the measurement instruments accurately represented the intended constructs.

Table 1 summarizes the structure of the research model by defining the major psychological variables and their roles. It clarifies how teacher burnout represents emotional challenges, emotion regulation functions as a protective psychological process, and learner engagement reflects students’ behavioural, emotional, and cognitive involvement in English learning. The inclusion of control variables strengthens the model by reducing the influence of external classroom factors.

**Table 1:** Description of study variables.

Variable	Role in model	Dimensions	Description
Teacher burnout	Independent variable	Emotional exhaustion, depersonalization, reduced personal accomplishment	Represents teachers’ psychological strain resulting from prolonged occupational demands
Emotion regulation	Mediating variable	Cognitive reappraisal, emotional control	Reflects teachers’ ability to manage and modify emotional responses in classroom situations
Learner engagement	Dependent variable	Behavioural, emotional, cognitive engagement	Indicates students’ participation, emotional involvement, and cognitive investment in learning
Teaching experience	Control variable	Years of teaching	Controls the influence of professional experience on classroom outcomes
Workload	Control variable	Teaching hours and responsibilities	Accounts for differences in occupational demands among teachers
Class characteristics	Control variable	Class size and learning context	Controls contextual differences affecting student engagement

Variables represent the proposed psychological model, including burnout, emotion regulation, and learner engagement constructs. Control variables were included to account for contextual differences among participants.

### 3.3 Data collection procedure and ethical considerations

Data collection was conducted during regular academic periods to ensure that responses reflected authentic classroom experiences. Teachers completed questionnaires related to their emotional experiences, burnout symptoms, and regulation strategies, whereas students provided information regarding their engagement in English learning activities. To connect teacher-level and student-level variables in the multilevel framework, teacher burnout and emotion regulation scores were linked to the corresponding student engagement scores for each teacher’s classroom. Student engagement was modeled as a student-level outcome (Level 1) with grand-mean centering for all predictors, which facilitates interpretation of within- and between-classroom effects. For the between-level analysis, student engagement scores were aggregated to the classroom level using the group mean. Aggregation was justified by an ICC of 0.28 and a high within-group agreement index (rwg = 0.84), indicating sufficient consensus among students within classrooms to support aggregation (James et al., 1984). These indices indicated sufficient between-classroom variability and within-classroom agreement to support the use of classroom-level aggregated engagement in the between-level component of the MSEM. Accordingly, aggregation was used only for the between-classroom representation of learner engagement, while individual student responses were retained for the within-classroom component of the multilevel model. Collecting data from both teachers and students reduced dependence on a single source of information and provided a broader understanding of classroom psychological processes. Prior to analysis, all responses were carefully screened for incomplete answers, unusual response patterns, and data quality issues. Minor missing responses were handled using appropriate statistical procedures to maintain the accuracy of the dataset (Cochran, 2026). Additional checks were performed to examine potential response bias because psychological variables are commonly assessed through self-report measures.

This study was conducted in accordance with the ethical standards of the institutional review board and the 1964 Helsinki Declaration and its later amendments. Ethical approval was obtained from the Research Ethics Committee of Lyuliang University (Approval Number: LLU-ER-2026-042). Informed consent was obtained from all participants prior to data collection, with separate consent forms for teachers and students. For student participants under 18 years of age, parental consent was obtained in addition to student assent. Participants were informed that their participation was voluntary, that they could withdraw at any time without penalty, and that their responses would be kept confidential and used only for research purposes.

### 3.4 Data analysis and structural model testing

Statistical analyses were conducted to examine the relationships among teacher burnout, emotion regulation, and learner engagement. Initially, descriptive statistics were calculated to identify overall patterns and variations within the study variables. Reliability analysis was performed using internal consistency indicators to ensure that all measurement scales demonstrated acceptable reliability. Confirmatory factor analysis (CFA) was conducted to evaluate the measurement structure and determine whether the proposed constructs were empirically distinct. The measurement model was evaluated using the following fit indices and criteria: comparative fit index (CFI ≥ 0.90), Tucker-Lewis index (TLI ≥ 0.90), root mean square error of approximation (RMSEA ≤ 0.08), and standardized root mean square residual (SRMR ≤ 0.08). For the multilevel CFA, we examined both within- and between-level factor structures. Discriminant validity was assessed by comparing the square root of the average variance extracted (AVE) for each construct with the correlations between constructs; discriminant validity is established when the square root of AVE exceeds the inter-construct correlations (Fornell and Larcker, 1981). Additionally, we examined the heterotrait-monotrait (HTMT) ratio of correlations, with values below 0.85 indicating adequate discriminant validity. Model fit was assessed using established indicators, including comparative fit index (CFI), Tucker–Lewis index (TLI), root mean square error of approximation (RMSEA), and standardized root mean square residual (SRMR).

Structural equation modeling (SEM) was then applied to test the proposed psychological framework. The analysis examined whether teacher burnout directly predicted learner engagement and whether emotion regulation acted as a mediating mechanism. Bootstrapping procedures were used to assess the significance of indirect effects and evaluate the statistical significance of the indirect association between teacher burnout and learner engagement through emotion regulation. Control variables, including teaching experience, workload, and classroom characteristics, were incorporated into the model to provide a more accurate estimation of the relationships among the main psychological variables (Silveira et al., 2026). This analytical strategy allowed the study to identify both the harmful effects of teacher burnout and the mediating role of emotional regulation in supporting learner engagement.

## 4 Results and discussion

### 4.1 Measurement model assessment and construct validation

Before evaluating the hypothesized structural relationships, the measurement model was examined to verify whether the observed indicators accurately represented the underlying psychological constructs. The results confirmed that teacher burnout, emotion regulation, and learner engagement demonstrated strong construct validity and reliability, providing a stable foundation for further structural analysis. The confirmatory factor analysis (CFA) for the measurement model demonstrated acceptable fit to the data: *χ*^2^ (87) = 214.56, *p* < 0.001, CFI = 0.95, TLI = 0.94, RMSEA = 0.058 (90% CI = 0.049–0.067), SRMR (within) = 0.039, SRMR (between) = 0.061. The multilevel CFA confirmed the hypothesized three-factor structure at both levels of analysis, with all factor loadings exceeding 0.70 (see Table 1). Discriminant validity was supported: the square root of AVE for each construct exceeded the correlations with other constructs, and all HTMT ratios were below 0.85. The highest correlation was between teacher burnout and emotion regulation (*r* = −0.39), and the HTMT ratio for this pair was 0.78, confirming that these constructs are empirically distinct despite their theoretical relationship. As presented in Table 1, all indicators showed satisfactory factor loadings, exceeding the recommended threshold, which indicates that each item contributed meaningfully to its respective construct. Teacher burnout was effectively represented through emotional exhaustion, depersonalization, and reduced professional efficacy, confirming its multidimensional psychological nature. Similarly, emotion regulation was adequately captured through cognitive reappraisal and expressive suppression, reflecting teachers’ capacity to manage emotional demands in classroom situations. Learner engagement showed strong representation through behavioural, emotional, and cognitive dimensions, supporting its conceptualization as a multidimensional learning process.

The reliability analysis further demonstrated acceptable internal consistency across all constructs. Teacher burnout achieved strong reliability (*α* = 0.89, CR = 0.91), while emotion regulation (*α* = 0.87, CR = 0.90) and learner engagement (*α* = 0.91, CR = 0.92) also showed high measurement consistency. Moreover, the AVE values exceeded the recommended level, confirming adequate convergent validity. These findings indicate that the measurement model successfully captured the intended psychological dimensions and was appropriate for testing the proposed relationships among teacher burnout, emotion regulation, and learner engagement.

Table 2 confirms that all psychological constructs demonstrated strong measurement quality. High factor loadings indicate that each indicator reliably represented its construct, while CR and AVE values support the reliability and validity of teacher burnout, emotion regulation, and learner engagement measures (Figure 1).

**Table 2:** Measurement model results for psychological constructs.

Construct	Indicator	Factor loading	Mean	SD	Cronbach’s *α*	CR	AVE
Teacher burnout	Emotional exhaustion	0.86	3.74	0.71			
	Depersonalization	0.82	3.41	0.68	0.89	0.91	0.63
	Reduced professional efficacy	0.84	3.58	0.66			
Emotion regulation	Cognitive reappraisal	0.85	3.96	0.62			
	Emotional control	0.88	4.02	0.59	0.87	0.90	0.61
Learner engagement	Behavioural engagement	0.87	3.89	0.64			
	Emotional engagement	0.90	3.95	0.61	0.91	0.92	0.65
	Cognitive engagement	0.86	3.82	0.67			

CR, Composite Reliability; AVE, Average Variance Extracted. Factor loadings above 0.70 indicate satisfactory indicator reliability.

**Figure 1:**
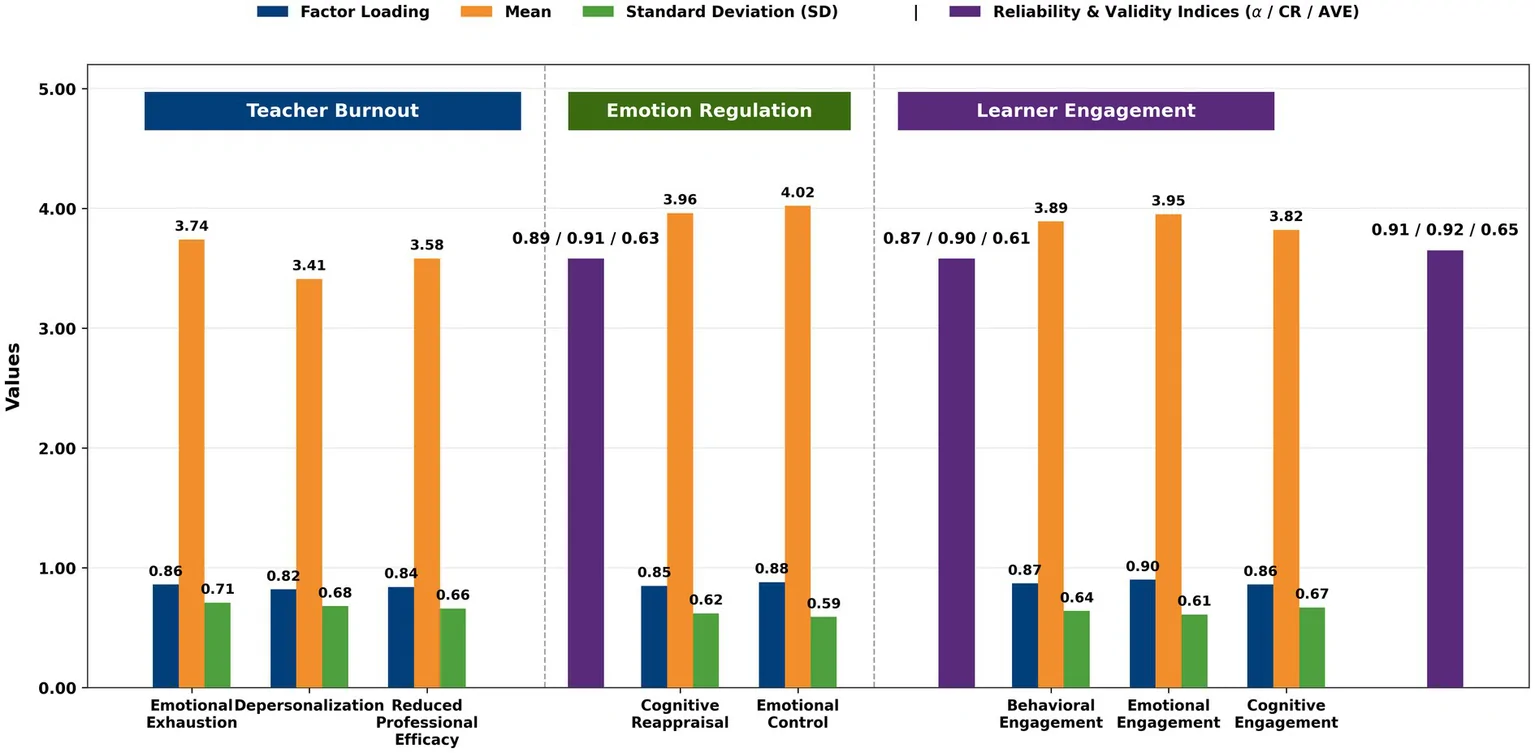
Measurement model validation of psychological constructs. Visual representation of the measurement model results for teacher burnout, emotion regulation, and learner engagement. The figure presents factor loadings, construct-level mean values, variability indicators, and reliability–validity statistics. All indicators demonstrated satisfactory factor representation, while Cronbach’s *α*, composite reliability (CR), and average variance extracted (AVE) values confirmed strong internal consistency and convergent validity of the psychological constructs. The findings support the suitability of the measurement model for examining the structural relationships among teacher burnout, emotional regulation, and learner engagement in English language classrooms.

### 4.2 Structural relationships among teacher burnout, emotion regulation, and learner engagement

Following the confirmation of the measurement model, the structural model was evaluated to examine the proposed relationships among teacher burnout, emotional regulation, and learner engagement. The analysis aimed to determine whether teachers’ psychological experiences significantly predicted students’ involvement in English language learning and whether emotional regulation functioned as a positive psychological resource within the classroom context. The results revealed that teacher burnout had a significant negative association with learner engagement (*β* = −0.36, *p* < 0.001). This finding indicates that higher levels of teacher burnout were linked with lower levels of student behavioural participation, emotional involvement, and cognitive investment in learning activities. Teachers experiencing greater emotional exhaustion, professional detachment, and reduced confidence in their teaching effectiveness may have fewer psychological resources available to provide encouragement, individualized support, and meaningful classroom interactions. In English language classrooms, where effective learning depends strongly on communication and active participation, reduced teacher emotional availability may negatively influence students’ willingness to engage with learning tasks. In contrast, emotional regulation demonstrated a significant positive effect on learner engagement (*β* = 0.42, *p* < 0.001). Teachers who were better able to manage and regulate their emotional responses appeared more capable of creating supportive and motivating classroom environments. Effective emotional regulation may indicate teachers to remain patient during challenging situations, respond constructively to student difficulties, and maintain positive teacher–student relationships. These findings suggest that emotional regulation is not simply a personal coping strategy but an important psychological resource that contributes to classroom effectiveness.

Overall, the structural model highlights the central role of teachers’ emotional processes in shaping student engagement. While burnout represents an emotional barrier that can reduce classroom involvement, strong emotional regulation may help teachers preserve supportive learning conditions. The findings provide further evidence that improving teacher psychological resources is essential for promoting meaningful engagement in English language classrooms (Table 3).

**Table 3:** Structural model path coefficients predicting learner engagement.

Hypothesized path	Estimate (*β*)	Standard error	Critical ratio	*p*-value	Decision	Effect size
Teacher burnout → Learner engagement	−0.36	0.05	−7.20	<0.001	Supported	Moderate
Teacher burnout → Emotion regulation	−0.31	0.06	−5.17	<0.001	Supported	Moderate
Emotion regulation → Learner engagement	0.42	0.04	10.50	<0.001	Supported	Strong
Teaching experience → Learner engagement	0.12	0.05	2.40	0.016	Supported	Small
Workload → Learner engagement	−0.14	0.05	−2.80	0.005	Supported	Small

Path coefficients represent standardized estimates from structural equation modeling. Significant paths indicate meaningful psychological relationships among study variables.

The structural results indicate that teacher burnout was negatively associated with learner engagement, whereas emotion regulation was positively associated with learner engagement., whereas emotional regulation functions as a positive classroom resource. Control variables showed smaller but significant contextual effects (Figure 2).

**Figure 2:**
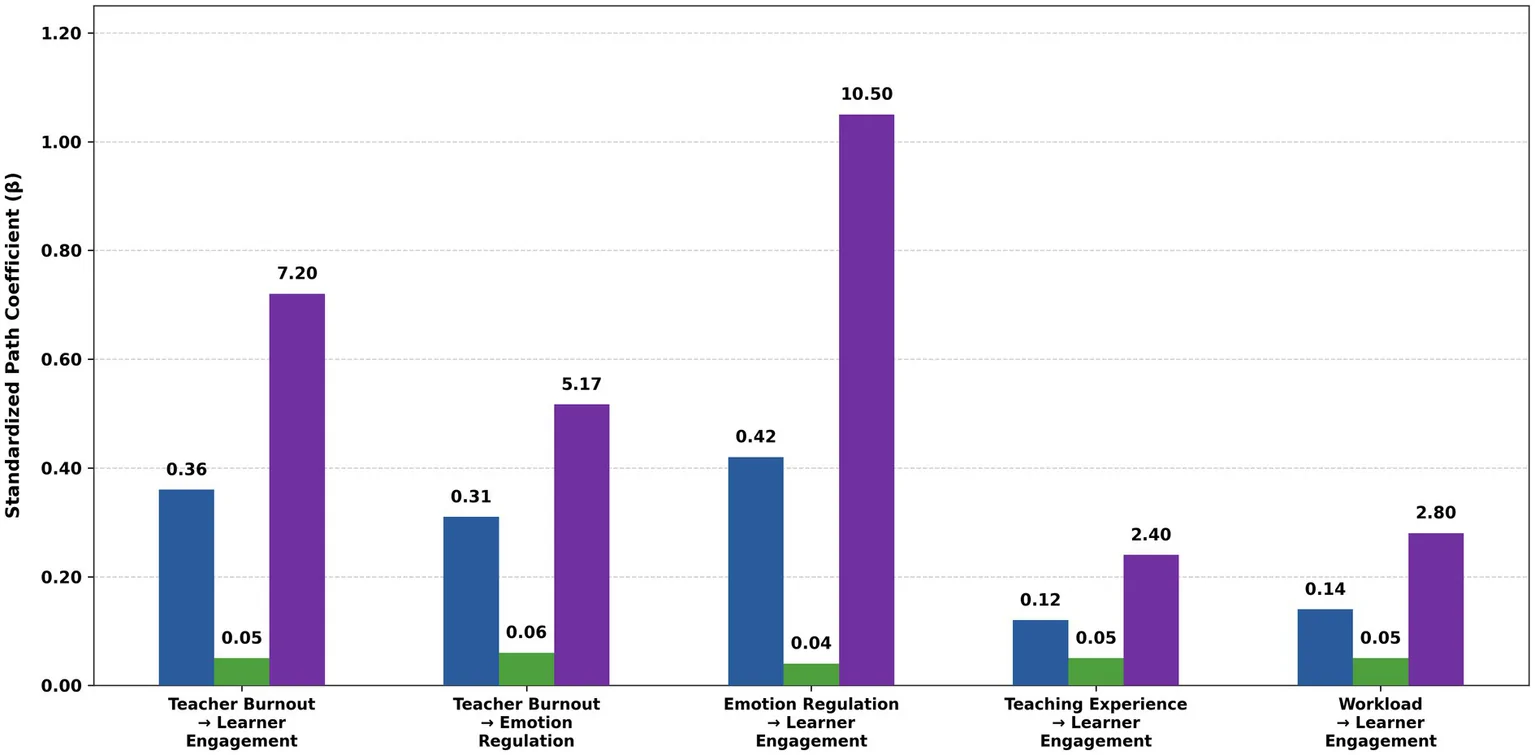
Structural model path coefficients predicting learner engagement. Structural model results illustrating the direct effects of teacher burnout, emotion regulation, and control variables on learner engagement. The bar chart presents standardized path coefficients (*β*), standard errors (SE), and critical ratios (CR) for each hypothesized relationship. Teacher burnout demonstrated a negative association with learner engagement, whereas emotion regulation showed a positive and stronger predictive effect. The results highlight the explanatory role of emotional regulation in maintaining student engagement despite teacher psychological strain. All estimated paths were statistically significant, supporting the proposed structural model of teacher emotional processes and learner engagement in English language classrooms.

### 4.3 Mediation analysis and psychological mechanism of emotion regulation

The final analysis examined whether emotion regulation statistically mediated the association between teacher burnout and learner engagement. The mediation results indicated that emotional regulation partially mediated the relationship between teacher burnout and learner engagement. To distinguish the mediation model coefficients from the direct effect model, we note that in the direct effect model (without the mediator), the total effect of burnout on engagement was −0.49 (*p* < 0.001). When emotion regulation was included as a mediator, the direct effect of burnout on engagement decreased to −0.36 (*p* < 0.001), while the indirect effect through emotion regulation was −0.13 (*p* < 0.001). This indirect effect was calculated as the product of the burnout → emotion regulation path (*β* = −0.31, *p* < 0.001) and the emotion regulation → engagement path (*β* = 0.42, *p* < 0.001), yielding −0.31 × 0.42 = −0.1302. The proportion mediated was calculated using the formula: indirect effect/(indirect effect + direct effect) = −0.13/(−0.13 + −0.36) = −0.13/−0.49 = 0.265, indicating that approximately 27% of the total effect of burnout on engagement operates through the emotion regulation pathway. The bootstrapped 95% confidence interval for the indirect effect (−0.19 to −0.08) did not contain zero, confirming the significance of the mediation pathway. Teachers experiencing higher burnout reported lower emotional regulation capacity, which subsequently predicted reduced learner engagement. This indirect pathway suggests that emotional regulation serves as a mediating mechanism that can support classroom engagement under stressful teaching conditions. Although burnout negatively affects teachers’ emotional resources, maintaining effective regulation strategies may reduce its impact on students’ learning experiences.

The mediation model provides an important theoretical contribution by identifying the emotional process through which teacher wellbeing influences classroom outcomes (Table 4).

**Table 4:** Mediation effect of emotion regulation between teacher burnout and learner engagement.

Mediation path	Direct effect	Indirect effect	Total effect	Bootstrapped 95% CI	Mediation type
Burnout → Emotion regulation → Engagement	−0.36	−0.13	−0.49	−0.19 to −0.08	Partial mediation

Additional mediation statistics:	
Indicator	Value
Bootstrap samples	5,000
Indirect effect significance	*p* < 0.001
Percentage mediated	27%
Variance explained (*R^2^* learner engagement)	46%

Confidence intervals were estimated using bootstrapping procedures. Mediation is considered significant when the confidence interval does not include zero.

The mediation findings indicate that emotion regulation partially explains the relationship between teacher burnout and learner engagement. Approximately 27% of burnout’s negative influence operates through reduced emotional regulation capacity, highlighting its mediating role (Figure 3).

**Figure 3:**
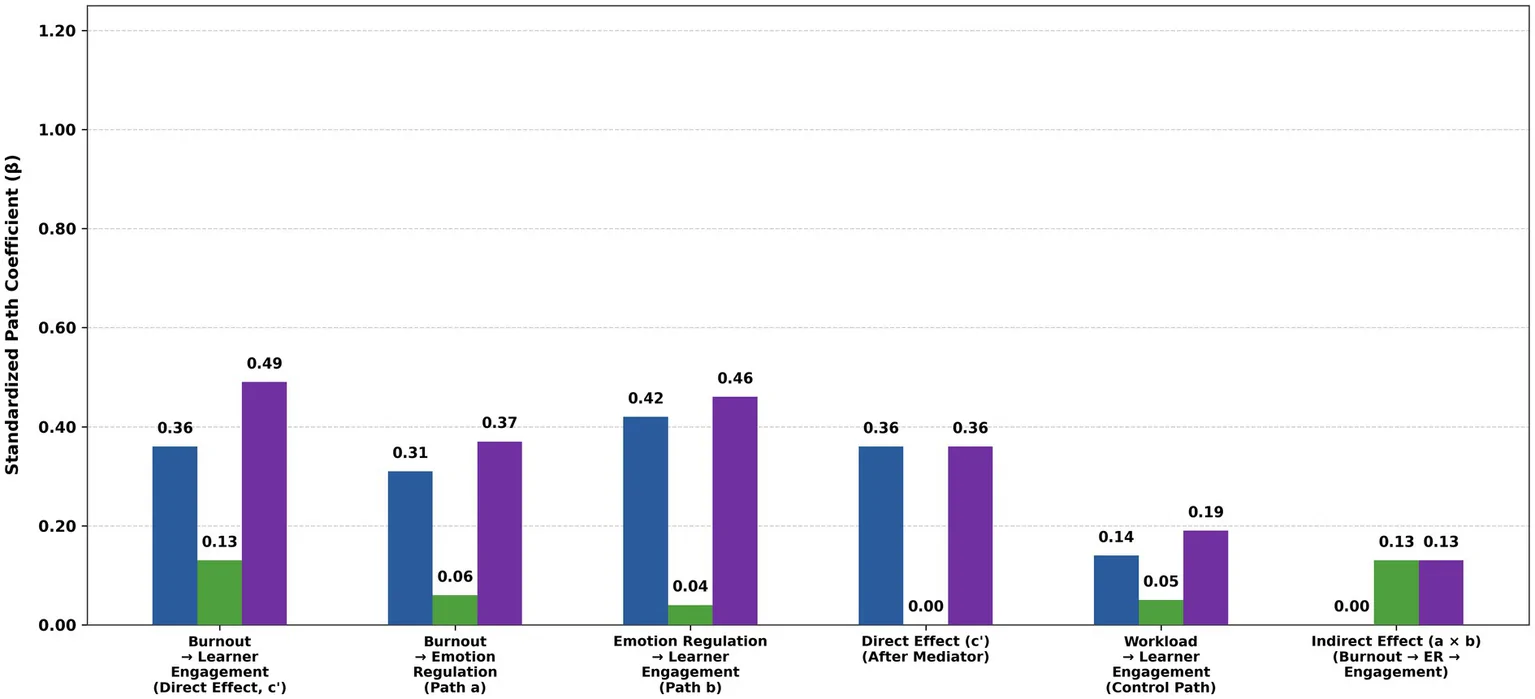
Mediation effect of emotion regulation between teacher burnout and learner engagement. Visualization of the mediation model demonstrating the indirect role of emotion regulation in the relationship between teacher burnout and learner engagement. The figure presents standardized path coefficients for the direct effect of teacher burnout on learner engagement, the pathway from burnout to emotion regulation, the effect of emotion regulation on learner engagement, and the resulting indirect and total effects. The mediation analysis indicates that emotion regulation partially mediates the negative influence of teacher burnout on learner engagement, with a significant indirect effect (*β* = −0.13, *p* < 0.001). The model was estimated using 5,000 bootstrap resamples, confirming the robustness of the mediation pathway. The findings indicate that emotion regulation represents a statistically significant mediating pathway in the association between teacher burnout and learner engagement.

### 4.4 Discussion

The findings of this study provide important evidence that teacher burnout should not be considered only as an individual occupational difficulty but also as a psychological factor that is associated with characteristics of the broader classroom learning environment. This conclusion aligns with and extends previous research by demonstrating that burnout is associated not only with teacher-level outcomes but also with differences in students’ learning experiences. For instance, while prior studies have documented that burnout is associated with reduced teaching effectiveness and lower job satisfaction (Li et al., 2025a; Solhi et al., 2026), the present findings suggest that burnout may also have cascading effects on the psychological quality of classroom environments. This is consistent with France and Harkins-Brown (2026) recent work on the “hidden cost” of teacher burnout, which argued that burnout represents a systemic educational challenge rather than merely an individual problem. The results demonstrate that teachers’ emotional experiences are closely connected with students’ engagement patterns, particularly in English language classrooms where communication, interaction, and emotional support are essential components of successful learning.

The magnitude of the direct association between burnout and engagement (*β* = −0.36) is comparable to findings reported in recent meta-analyses. For example, a meta-analysis by Madigan and Kim (2021) found an average correlation of r = −0.30 between teacher burnout and various student outcomes, suggesting that the present finding is consistent with the broader literature. However, the present study extends this meta-analytic evidence by demonstrating that this association remains significant when controlling for contextual factors such as workload and teaching experience, and by examining it specifically within English language teaching contexts where emotional demands may be particularly high.

The stronger effect of emotion regulation on engagement (*β* = 0.42) compared to the effect of burnout (*β* = −0.36) is noteworthy. This pattern aligns with the theoretical proposition that teachers’ regulatory capacities may be more proximal predictors of classroom quality than burnout itself (Jennings and Greenberg, 2009). In other words, while burnout represents a distal risk factor, emotion regulation represents a more immediate psychological resource that directly shapes teachers’ ability to create supportive learning environments. This distinction has important implications for intervention design, suggesting that efforts to enhance emotion regulation may be particularly effective even when burnout levels remain elevated.

The negative association between teacher burnout and learner engagement indicates that prolonged emotional exhaustion, reduced professional efficacy, and psychological distancing may weaken teachers’ ability to maintain supportive relationships, provide meaningful feedback, and encourage active student participation. The findings further highlight the significant role of emotion regulation as a key psychological mechanism. This finding is consistent with the theoretical predictions of Gross's (1998) emotion regulation model and with empirical research demonstrating that cognitive reappraisal, in particular, is associated with more adaptive coping in occupational settings (Wang et al., 2026). However, the present study extends this research by examining emotion regulation specifically within English language teaching contexts. The stronger effect of emotion regulation on engagement (*β* = 0.42) compared to the negative effect of burnout (*β* = −0.36) suggests that teachers’ regulatory capacities may be particularly important in language classrooms, where communication, interaction, and emotional exchange are central to the learning process. Teachers who demonstrate stronger emotional regulation abilities are more capable of managing classroom pressures, controlling negative emotional responses, and maintaining constructive interactions with students. Rather than eliminating professional challenges, effective emotion regulation appears to help teachers respond to stressful situations more adaptively and preserve a positive classroom atmosphere. This suggests that emotional regulation serves as an important pathway through which teachers’ psychological resources influence student learning experiences.

The mediation results extend previous research by showing that emotion regulation represents a potential explanatory pathway between teacher wellbeing and learner engagement. The finding that approximately 27% of burnout’s negative effect on engagement operates through reduced emotion regulation capacity provides empirical support for the theoretical model proposed by Wang et al. (2026) and aligns with the broader literature on emotional resources as mechanisms linking occupational stress to relational outcomes. This mediation pathway is consistent with recent studies showing that emotional regulation mediates the relationship between various stressors and teaching outcomes (Guzmán-Martínez et al., 2026; Research and Teaching, 2026; Quarterly, 2026). However, unlike previous research that has primarily examined emotion regulation as an outcome of stress, the present study positions it as a mediating mechanism that statistically accounts for part of the association between teacher burnout and learner engagement”. This is much safer scientifically. While burnout creates emotional barriers that may reduce classroom involvement, teachers with stronger regulatory abilities may overcome these difficulties and continue to support students’ academic and emotional needs. This finding contributes to educational psychology by emphasizing that classroom effectiveness depends not only on instructional competence but also on teachers’ internal emotional processes.

#### 4.4.1 Theoretical contributions to emotion regulation research

The finding that approximately 27% of burnout’s negative effect on engagement operates through reduced emotion regulation capacity provides empirical support for the resource depletion model proposed within COR theory (Hobfoll, 1989). When teachers experience burnout, their emotional resources are depleted, which compromises their ability to engage in the cognitive reappraisal and emotional control strategies necessary for effective teaching. This resource depletion then manifests in reduced classroom engagement among students. This interpretation is consistent with Wang et al.'s (2026) recent finding that emotion regulation mediated the relationship between emotional experiences and engagement in multilingual learning contexts, though the present study extends this work by examining the specific pathway from burnout to engagement through regulation.

The partial nature of the mediation—with 73% of burnout’s effect remaining direct—suggests that emotion regulation is an important but not exclusive mechanism. Other pathways not examined in this study may also transmit burnout’s effects to students. For instance, burnout may directly reduce instructional quality, limit teachers’ ability to provide timely feedback, or reduce the frequency of positive teacher-student interactions independent of emotion regulation (Ma and Li, 2026). Future research should examine these alternative pathways and test whether they operate in parallel with or in addition to the emotion regulation mechanism identified here.

From a practical perspective, educational institutions should adopt a more comprehensive approach to teacher development.

It is important to interpret the mediation findings with attention to the multilevel structure of the data. Because teacher burnout and emotion regulation were measured at the teacher level, and student engagement at the student level, the mediation pathway operates across levels. This means that the observed indirect effect reflects how between-teacher differences in burnout relate to between-teacher differences in emotion regulation, which in turn relate to between-teacher differences in average student engagement. The within-classroom variability in engagement (approximately 72% of the total variance) was not the focus of the present mediation model. Future research may benefit from examining cross-level interactions, such as whether the relationship between teacher burnout and student engagement varies depending on individual student characteristics (e.g., motivation, language proficiency, or prior achievement).

These implications are consistent with recent calls for integrating psychological support into teacher professional development frameworks (Dou et al., 2026; Fu et al., 2026). However, it is important to note that the cross-sectional design of this study precludes causal conclusions about intervention effects. While the findings suggest that emotion regulation training and burnout prevention programs may be beneficial, these recommendations should be treated as theoretically grounded suggestions that require empirical validation through experimental or longitudinal research (France and Harkins-Brown, 2026). Professional training programs could consider incorporating emotional regulation skills, stress management techniques, and psychological support alongside traditional instructional training. Creating supportive organizational environments may help reduce burnout risks and strengthen teachers’ emotional resources. By investing in teachers’ psychological wellbeing, such organizational investments may contribute to healthier classroom relationships and learner engagement, subject to validation in longitudinal or experimental research. Several alternative explanations for the observed relationships should be acknowledged. Although the mediation model positions emotion regulation as the pathway through which burnout affects engagement, it is possible that other unmeasured variables, such as classroom climate, institutional support, or teacher-student relationship quality, also play important roles in this process. For instance, supportive leadership and positive organizational culture have been shown to buffer the effects of burnout (Boroughani et al., 2025; France and Harkins-Brown, 2026), suggesting that contextual factors may moderate the relationships examined in this study. Moreover, the direction of effects cannot be definitively established due to the cross-sectional design; it is possible that low student engagement contributes to teacher burnout, creating a bidirectional relationship that was not captured in the present analysis. Future longitudinal research is needed to disentangle these directional effects and examine potential reciprocal influences over time.

### 4.5 Recommendations for future research

The present findings suggest several directions for future investigation. First, longitudinal studies are needed to establish the temporal ordering of the burnout-regulation-engagement pathway. While the present study is grounded in a theoretically derived mediation model, cross-sectional data cannot confirm that burnout precedes reductions in emotion regulation, which in turn precede declines in engagement. Experience sampling methods (ESM) or daily diary studies could capture the within-person fluctuations in burnout, emotion regulation, and engagement across teaching days, providing stronger evidence for causal processes (Burić and Macuka, 2018). Second, intervention studies that experimentally enhance teachers’ emotion regulation skills and track subsequent changes in burnout and student engagement would provide more definitive evidence regarding causality and practical effectiveness. Third, future research should examine potential moderators of the mediation pathway, such as school leadership support, collegial relationships, and classroom characteristics (e.g., class size, student age, language proficiency levels), which may strengthen or weaken the observed relationships. Fourth, cross-cultural comparisons would help determine whether the mediation model operates similarly across different educational systems and cultural contexts, given that cultural norms regarding emotional expression and regulation may influence the effectiveness of specific strategies (Matsumoto et al., 2008).

## 5 Conclusion

This study provides a comprehensive understanding of how teachers’ emotional experiences influence learner engagement in English language classrooms. Moving beyond the traditional view of teacher burnout as an individual occupational problem, the findings indicate that burnout is associated with broader psychological processes that relate to classroom interactions and students’ learning experiences. The results highlight that teachers’ emotional states are closely connected with students’ behavioural participation, emotional involvement, and cognitive investment in learning activities. In particular, the study shows that higher levels of emotional exhaustion, professional detachment, and reduced feelings of effectiveness are associated with lower student engagement within the classroom environment.

A major contribution of this research is the identification of emotion regulation as a key psychological mechanism underlying the relationship between teacher burnout and learner engagement. The findings indicate that emotional regulation is not merely associated with individual coping but also appears to relate to classroom resources and educational outcomes. Teachers who are better able to manage emotional demands, reinterpret stressful situations, and control emotional responses appear more capable of maintaining supportive relationships, encouraging student participation, and creating positive learning environments. This suggests that teachers’ internal emotional resources play a significant role in shaping the psychological quality of classroom interactions.

The study also provides theoretical advancement by integrating emotion regulation theory with educational psychology perspectives to explain how teacher wellbeing influences student learning processes. Previous research has frequently examined burnout in relation to teachers’ professional outcomes; however, this study extends existing knowledge by demonstrating the pathway through which teacher emotional experiences influence learner engagement. The mediation findings suggest that burnout is related to lower student engagement both directly and indirectly through its association with reduced emotional regulation capacity. This provides a more complete explanation of the psychological dynamics operating within English language classrooms.

### 5.1 Practical implications for teacher education and policy

The findings of this study have several practical implications for teacher education, professional development, and educational policy. First, teacher training programs should incorporate explicit instruction in emotion regulation strategies, particularly cognitive reappraisal, as part of their core curriculum. Rather than treating emotional skills as peripheral to content knowledge and pedagogical methods, teacher educators should recognize emotional competence as a foundational professional competency. Second, school administrators should create organizational conditions that support teachers’ emotional wellbeing, including manageable workloads, collegial support structures, and access to psychological resources. Third, educational policymakers should consider integrating emotional support systems into teacher evaluation and professional development frameworks, recognizing that teachers’ emotional resources are essential for creating engaging learning environments. Fourth, as the present study was conducted in English language teaching contexts, language teacher education programs may benefit from particular attention to emotion regulation, given the high emotional and interpersonal demands associated with language instruction (Gkonou and Mercer, 2017). The integration of these practical recommendations, while requiring further empirical validation through intervention studies, offers a theoretically grounded pathway toward improving both teacher wellbeing and student engagement in educational settings.

From a practical perspective, the findings emphasize the need for educational institutions to adopt a broader approach to teacher development and support. Traditional professional development programs often focus primarily on instructional methods, curriculum planning, and assessment practices. However, the present findings suggest that teachers’ emotional competencies should receive equal attention. Training programs that strengthen emotional regulation skills, stress management strategies, and adaptive coping mechanisms may help teachers manage occupational pressures more effectively. Such interventions may not only improve teacher wellbeing but also contribute to more engaging and supportive learning experiences for students.

Educational policymakers and institutional leaders should consider integrating psychological support systems into teacher development frameworks. Creating supportive working environments, reducing excessive occupational demands, and providing opportunities for emotional skill development may reduce burnout risks and enhance teachers’ ability to maintain effective classroom relationships. In English language education, where communication and interpersonal connection are fundamental to learning success, strengthening teachers’ emotional resources may have particularly meaningful effects on student engagement.

Several limitations should be acknowledged when interpreting the findings. First, the cross-sectional design prevents causal conclusions about the direction of relationships among burnout, emotion regulation, and engagement. While the structural model is theoretically grounded, alternative model specifications—such as engagement influencing burnout or burnout influencing emotion regulation in the opposite direction—cannot be ruled out. Second, the reliance on self-report measures raises concerns about common method variance, although the use of multiple sources (teachers and students) partially mitigates this concern. Third, the data were collected from a single geographical region, which limits the generalizability of findings to other cultural and educational contexts. Fourth, the study did not examine potential moderators, such as teaching experience, classroom size, or institutional support, which may influence the strength or direction of the observed relationships. Future research may examine these relationships using longitudinal designs to better understand how teacher burnout and emotional regulation develop over time. Experimental studies evaluating the effectiveness of emotional regulation interventions could provide stronger evidence regarding practical applications. Additionally, future research may explore how institutional culture, leadership support, technological changes, and cultural differences influence the relationship between teacher emotions and learner engagement.

Overall, this study demonstrates that improving educational outcomes requires attention not only to teaching practices but also to the psychological experiences of teachers. Teacher burnout represents a significant challenge, but emotion regulation provides a valuable pathway for protecting classroom engagement and promoting healthier learning environments. By recognizing teachers as emotional as well as instructional agents, educational systems can develop more sustainable approaches that support both teacher wellbeing and meaningful student engagement in English language classrooms.

## Data Availability

The original contributions presented in the study are included in the article/supplementary material, further inquiries can be directed to the corresponding author.

## References

[ref1] Boroughani T. Rivera H. Xodabande I. (2025). Teaching behind the screen: emotional labor of Iranian language teachers in online classes. In: Nazari, M. (eds.) Language Teachers’ Emotional Dynamics in Technology-Based Contexts. Springer, Singapore. 141–162. doi: 10.1007/978-981-95-2569-0_8

[ref2] Burić I. Macuka I. (2018). Self-efficacy, emotions and work engagement among teachers: a two-wave cross-lagged analysis. J. Happiness Stud. 19, 1917–1933. doi: 10.1007/s10902-017-9903-4

[ref3] Cochran T. C. (2026). Social Change in Industrial Society: Twentieth-Century America. Taylor & Francis.

[ref4] Deci E. L. Ryan R. M. (2000). The "what" and "why" of goal pursuits: human needs and the self-determination of behaviour. Psychol. Inq. 11, 227–268. doi: 10.1207/S15327965PLI1104_01, 36589895

[ref5] Dou D. Huang C. Li J. Shi J. (2026). Trends, Challenges, and Future Directions in research on Social-Emotional Learning Programs and Teacher Wellbeing. Applied Research in Quality of Life, Springer. 1–24.

[ref6] Fornell C. Larcker D. F. (1981). Evaluating structural equation models with unobservable variables and measurement error. J. Mark. Res. 18, 39–50. doi: 10.1177/002224378101800104, 33691717

[ref7] France M. Harkins-Brown A. (2026). The hidden cost of teacher burnout: implications for student learning and wellbeing. Taylor & Francis 99, 164–172. doi: 10.1080/00098655.2026.2661785

[ref8] Fredricks J. A. Blumenfeld P. C. Paris A. H. (2004). School engagement: potential of the concept, state of the evidence. Rev. Educ. Res. 74, 59–109. doi: 10.3102/00346543074001059, 38293548

[ref9] Fu Y. Lin Z. Zeng G. Lu X. (2026). Between resilience, academic engagement, and English academic achievement among children in rural regions: the mediating effects of academic emotions. Spring 45. doi: 10.1007/S12144-025-08846-0

[ref10] Gan L. Zhu S. Wang L. (2026). Autonomy-supportive teaching, positive academic emotions, and agentic engagement in EFL contexts. BMC Psychol. 14. doi: 10.1186/S40359-026-04531-4, 41981642 PMC13188635

[ref11] Gkonou C. Mercer S. (2017). “Understanding emotional and social intelligence among English language teachers,” in Emotions in Second Language Teaching, ed. Martínez Agudo J. d. D. (UK: Springer), 99–116.

[ref12] Gross J. J. (1998). The emerging field of emotion regulation: an integrative review. Rev. Gen. Psychol. 2, 271–299. doi: 10.1037/1089-2680.2.3.271

[ref13] Gross J. J. John O. P. (2003). Individual differences in two emotion regulation processes: implications for affect, relationships, and wellbeing. J. Pers. Soc. Psychol. 85, 348–362. doi: 10.1037/0022-3514.85.2.348, 12916575

[ref14] Guzmán-Martínez P. Varela J. J. Hidalgo-Canales R. Valdivia M. B. Cortés J. (2026). “Teacher emotions: a view from the perspective of teacher wellbeing and resilience,” in Springer, (USA), 107–125.

[ref15] Hatfield E. Cacioppo J. T. Rapson R. L. (1994). Emotional contagion. UK: Cambridge University Press.

[ref16] Hobfoll S. E. (1989). Conservation of resources: a new attempt at conceptualizing stress. Am. Psychol. 44, 513–524. doi: 10.1037/0003-066X.44.3.513, 2648906

[ref17] Hochschild A. R. (1983). The Managed Heart: Commercialization of Human Feeling. California: University of California Press.

[ref18] James L. R. Demaree R. G. Wolf G. (1984). Estimating within-group interrater reliability with and without response bias. J. Appl. Psychol. 69, 85–98. doi: 10.1037/0021-9010.69.1.85

[ref19] Jennings P. A. Greenberg M. T. (2009). The prosocial classroom: teacher social and emotional competence in relation to student and classroom outcomes. Rev. Educ. Res. 79, 491–525. doi: 10.3102/0034654308325693, 38293548

[ref20] Lagos N. Martín S. Kralova Z. Li G. Chen C. Guo Z. (2026). From foreign language classroom anxiety to English learning engagement: the roles of cognitive reappraisal and expressive suppression. Frontiersin.Org 17. doi: 10.3389/FPSYG.2026.1754113/FULL PMC13008866 41884541

[ref21] Li L. Steins G. Mosley K. C. Zeng Y. (2025). Burnout Among Chinese EFL University Instructors. Books.Google.Com.

[ref22] Liu G. L. Ghorbandordınejad F. Zhao X. Esirgapov M. N. (2026). Social Support, Resilience, and (AI-Mediated) Informal Digital Learning of English Among Multilingual Learners in Uzbekistan. Wiley Online Library.

[ref23] Ma L. Li G. (2026). Teacher emotional support and academic engagement in university English learning: the mediating effects of self-efficacy and positive emotions. Journals.Sagepub.Com 16. doi: 10.1177/21582440261416068

[ref24] Madigan D. J. Kim L. E. (2021). Towards an understanding of teacher attrition: a meta-analysis of burnout, job satisfaction, and teachers' intentions to quit. Teach. Teach. Educ. 105:103425. doi: 10.1016/j.tate.2021.103425

[ref25] Maslach C. Jackson S. E. Leiter M. P. (1996). Maslach Burnout Inventory Manual. 3rd Edn. Scarecrow: Consulting Psychologists Press.

[ref26] Matsumoto D. Yoo S. H. Nakagawa S. (2008). Culture, emotion regulation, and adjustment. J. Pers. Soc. Psychol. 94, 925–937. doi: 10.1037/0022-3514.94.6.925, 18505309

[ref27] Quarterly A. R.-T. (2026). TESOL practicum boredom: causes, consequences, and coping strategies among pre-service teachers. Wiley Online Library 60, 167–217. doi: 10.1002/TESQ.70033

[ref28] Research and Teaching (2026). Relations between teacher—student relationship quality, emotional engagement, and writing performance. Journals.Sagepub.Com 30, 2607–2630. doi: 10.1177/13621688231199821

[ref29] Silveira R. M. F. McManus C. da Silva I. J. O. (2026). Animal production under climate change: a global scientometric analysis of research structure, thematic evolution, and knowledge gaps. Trop. Anim. Health Prod. 58. doi: 10.1007/S11250-026-05071-0, 42207432 PMC13219208

[ref30] Solhi M. Elahi Shirvan M. Taherian T. (2026). Self-efficacy and learning engagement in EFL context: a moderated mediation model of enjoyment, anxiety, and boredom. Elsevier 11. doi: 10.1186/S40862-026-00388-7 41679149

[ref31] Sutton R. E. Wheatley K. F. (2003). Teachers' emotions and teaching: a review of the literature and directions for future research. Educ. Psychol. Rev. 15, 327–358. doi: 10.1023/A:1026131715856, 41886696

[ref32] Wang Y. Liu W. Li S. (2026). The mediating role of emotion regulation in the link between enjoyment, anxiety, and emotional disengagement in multilingual learning: a response surface analysis. Taylor & Francis. doi: 10.1080/01434632.2026.2679605

[ref33] Wei Y. Wang X. (2026) Perceived teacher support on junior high school students' emotional engagement in English class: based on a structural equation modeling analysis.

[ref34] Xu G. Haratyan F. Tian H. (2026). Teacher emotional intelligence and learner engagement: a cross-sectional analysis in Iranian EFL contexts. Journalapes.Com 16, 1–17. doi: 10.3389/FPSYG.2025.1715266/FULL

[ref35] Yao N. Yang L. Mutlu A. K. (2026). Apprehension, self-efficacy and academic engagement in technology-mediated EFL classrooms: a cross-sectional study of Mongolian trilingual learners. Wiley Online Library 61. doi: 10.1111/EJED.70397

